# Selections and Abstracts

**Published:** 1908-12

**Authors:** 


### SELECTIONS *and* ABSTRACTS

### FREMONT COUNTY (WYOMING) MEDICAL ASSOCIATION—RESOLUTION ON DEAD-BEATS.

The officers and members of the Fremont County Medical Association, being in regular session, deeming it wise to take action in regard to persons who are "Dead-Beats" and others who are slow pay, in order to receive compensation for their services and to discourage the public from employing one physician while already indebted to another who is a member of this association, and

Whereas, The members of this association have suffered in the past from wilful neglect of those who are able, but unwilling to pay for the services rendered by physicians,

And whereas, Certain persons have patronized one physician as long as credit was extended, and then changed to another, and so on, as long as another was to be had, or credit could be obtained.

And whereas, In order to ingratiate themselves into the good graces and confidence of the physician subsequently called, they have resorted to false promises and villification of other members of the profession,

And whereas, The members of the profession have been put to a great deal of expense, as livery hire, cost of drugs, surgical dressings, etc., and have been humiliated by the ingratitude and discourtesy of said persons, by misrepresentation and calumny,

And whereas, Such conditions are likely to lead to discord and misunderstanding between members of this association, therefore,

Be it resolved, That the members of this association be instructed to present the names of such persons to the secretary, who shall read them at the next regular meeting, and furnish each member with a copy of the same,

And be it Resolved, That for reasons already set forth, every member of this association, shall refuse to extend any credit or services whatever, to such person or persons, or his or her family but upon being called, shall demand his fees in advance, and continue to do so until said person shall have made satisfactory settlement with the physician to whom he or she is indebted and his or her name withdrawn from the list.

And Be it Further Resolved, That when legal measures are instituted for the collection of such accounts against such person or persons, by any member of this association, each and every member of same shall render all aid possible in prosecution of such suit, and the collection of the same,

Further, That violations of these tenets shall be reported to the censors who shall investigate the same, and if found worthy, prefer charges.

Further, That such list shall be known as the "Black List," and that this resolution be spread upon the minutes of this meeting, and that a copy be sent to every member of this association. —*Western Medical Review.*

### THE FIRST OPERATION FOR APPENDICITIS.

The question is often asked, "When was the first operation for appendicitis performed?" We have been asked it this week almost exactly in these words. The answer must depend greatly on what is meant by operations for appendicitis. If we may include among such operations the evacuation of an abscess resulting from an attack of appendicitis we must go back a very long way indeed. Doubtless many such abscesses were opened ages before any record of such an operation was made. Aretaeus, who flourished some 50 years before the commencement of the Christian era, says: "I once made an incision into an abscess in the colon on the right side near the liver and much pus gushed out." This may have been an appendix abscess, but he goes on to say that much pus was evacuated also with the urine, so that we cannot be sure that it was not a pyonephrosis. Here and there through the following centuries we find cases recorded which are fairly certainly examples of incision of an appendix abscess, but it was not until 1759 that we meet with an operation for abscess which was definitely shown to be due to disease of the vermiform appendix; in that year Mestivier incised an abscess on the right side of the abdomen near the umbilicus and much pus was evacuated. The wound healed but the patient died before long and at the necropsy a pin was found in the appendix with many signs of inflammation. Seven years later Lamotte described a large foecal concretion in the appendix, but the discovery was only made post mortem. In 1848 Hancock reported the opening of an abscess immediately above Poupart's ligament on the right side and later two foecal concretions came away. The incision was made early, even before fluctuation could be detected. In 1867 Parker published four similar cases and from that time the opening of abscess in the right iliac fossa became less rare. The earliest suggestion to remove the appendix appears to have been made by Fenwick in 1884, and this operation was performed by Kronlein in the same year. He opened the abdomen of a boy aged 17 years who had general peritontitis and ligatured and removed the perforated appendix. Some temporary improvement followed, but death occurred three days after the operation. Symonds, in 1885, removed a concretion from an appendix without opening the peritoneal cavity. The first successful operation for removing the appendix was performed by Morton in 1887 and from that time the operation has become common. We have then answered the question, "When was the first operation for appendicitis performed?" by showing that appendix abscesses have been opened many centuries ago; that Hancock, in 1848, incised an appendix abscess before fluctuation could be felt; that Kronlein, in 1884, removed a perforated appendix but the patient died; and that Morton, in 1887, had the first successful case of appendicetomy.—*Lancet.*

